# Effects of Visceral Fat Accumulation Awareness on a Web-Based Weight-Loss Program: Japanese Study of Visceral Adiposity and Lifestyle Information—Utilization and Evaluation (J-VALUE)

**DOI:** 10.1155/2013/473764

**Published:** 2013-04-24

**Authors:** Naoki Sakane, Seitaro Dohi, Koichi Sakata, Shin-ichi Hagiwara, Toshihisa Morimoto, Takanobu Uchida, Mitsuhiro Katashima, Yoshiko Yanagisawa, Takeshi Yasumasu, J-VALUE Study Group

**Affiliations:** ^1^Division of Preventive Medicine, Clinical Research Institute, National Hospital Organization Kyoto Medical Center, 1-1 Mukouhata-cho, Fukakusa, Fushimi-ku, Kyoto 612-8555, Japan; ^2^Health Management Department, Mitsui Chemicals Inc., Shiodome City Center 1-5-2, higashi-Shinbashi, Minato-ku, Tokyo 105-7117, Japan; ^3^Nippon Steel & Sumitomo Metal Corporation, 5-33 Kitahama 4-Chome, Chuo-ku, Osaka 541-0041, Japan; ^4^EG Health Care Center, Honda Engineering Co., Ltd, 6-1 Hagadai, Haga-machi, Haga-gun, Tochigi 321-3395, Japan; ^5^Kao Corporation, 2-1-3 Bunka, Sumida-ku, Tokyo 131-8501, Japan

## Abstract

A reduction of visceral fat is important for improvement of metabolic risk. This study was designed to compare the effects of a web-based program alone or together with measurement and self-awareness of accumulated visceral fat in Japanese workers. A new noninvasive device to measure visceral fat accumulation was introduced, and efficacy on weight-loss and improvement of healthy behaviors were examined. This study was conducted according to Helsinki declaration and approved by the ethical committee of Japan Hospital Organization, National Kyoto Hospital. Two-hundred and sixteen overweight and obese males with BMI of more than 23 participated from 8 healthcare offices of 3 Japanese private companies. Subjects were randomly allocated into control group, Web-based weight-loss program (Web), or Web + Visceral fat measurement group (Web + VFA). Eighty-one percent of participants completed the study. Reductions of body weight, waist circumference, and BMI were the largest in Web + VFA group, and the differences between groups were significant by ANOVA. Improvements of healthy behaviors were the largest in Web + VFA group, and the differences of healthy eating improvement scores between Web + VFA and control groups were significant. Our findings suggest that measurement and awareness of visceral fat are effective in weight reduction in overweight and obese males in the workplace.

## 1. Introduction 

Type 2 diabetes mellitus (T2DM) is rapidly becoming one of the major health issues of the 21st century [1, 2]. A recent survey performed by the Ministry of Health, Labor and Welfare projected that approximately 8.9 million people have diabetes and another 13.2 million people are at high risk for the disease in Japan [3, 4]. Obesity and upper body/abdominal fat distribution are associated with increased risk of type 2 diabetes, hypertension, hyperlipidemia [5, 6], heart disease, [7] and overall mortality [8]. Body fat distribution is recognized as a predictor of the metabolic complications of obesity [9]. Indeed, visceral adipose tissue was found to be more strongly linked with the risk of hyperlipidemia [10, 11], insulin resistance [12–14], and diabetes [15, 16] than subcutaneous fat. In these studies, computed tomography (CT) scans at the umbilical level were used for the assessment of visceral fat accumulation (VFA). This method is not cost-effective, however, and leads to radiation exposure. A new noninvasive medical device that measures visceral fat by area has recently been under development [17]. Using a Web-based weight-loss program, the aim of this study was to compare the effects of information on visceral fat accumulation (VFA) on waist circumference (WC), body weight (BW), BMI, and healthy workplace behavior.

## 2. Subjects and Methods

### 2.1. Ethics Approval

The study protocol was approved by the Ethics Committee of the National Hospital Organization Kyoto Medical Center (Approval no. 09-34). All subjects gave their written informed consent before the start of the study.

### 2.2. Design

The present study was an assessor-blinded Randomized Clinical Trial (RCT) design with a 12-week followup. Subjects were allocated to 1 of the following 3 groups: (1) control, (2) Web-based weight-loss program (Web), and (3) Web plus visceral fat measurement and education focused on increased health risks associated with visceral fat accumulation (Web + VFA).

### 2.3. Recruitment of the Subjects

Overweight and obese subjects were recruited from 8 health facilities run by 3 companies between July 2009 and March 2011. Pamphlets, posters, and other recruiting media that were specifically targeted at subjects of various age groups were prepared. Regional medical staff freely selected appropriate media based on the characteristics of their subjects. Participants in this study comprised men and women, 20–65 years of age who were either overweight (BMI: 23–24.9) or obese (BMI ≥25) according to WHO Regional Office for Western Pacific (WPRO) criteria, and had given written informed consent. Individuals were excluded from the study if they had steel-based medical implants such as coils and/or plates, were pregnant or planning a pregnancy, or did not receive approval from medical doctors to participate in the study. 

### 2.4. Sample Size

The primary outcome of the present study was change in WC (cm) at 12 weeks. In order to detect a 3.4 cm reduction in WC with 90% power at a significance level of 0.05 (two-sided), more than 174 participants with 58 in each study arm were required, assuming an expected dropout rate of 0.18.

### 2.5. Randomization

Subjects were allocated to groups as follows by a quasirandomized design based on their date of birth: control, born between the 21st and the 31st of the month; Web, between the 11th and the 20th; or Web + VFA, between the 1st and the 10th. Therefore, the division of subjects was not influenced by age, sex, or any other factor.

## 3. Measures

### 3.1. Primary Outcome Measures

Waist circumference at the umbilical level was measured with nonstretchable tape in the late expiration phase while standing.

### 3.2. Secondary Outcome Measures

Height and weight were measured in a standing position. The subjects were weighed in light clothing using a calibrated scale, and BMI was calculated as kg/m^2^. Measurements were performed prior to the start of the study and at every 4 weeks after starting the intervention. Age and other demographic variables were collected, as well as variables including readiness to engage in physical activities and healthy eating habits. Achievement of readiness was assessed by a self-reported questionnaire at baseline and again at 12 weeks according to the following four stages reported in “Stages of Change Model” by Prochaska and DiClemente: (1) precontemplation, (2) contemplation, (3) preparation, and (4) action/maintenance. The scores were self-recorded using a special website designed for this purpose.

### 3.3. Intervention

The 12-week intervention program was conducted with a focus on modification of physical activities and healthy eating habits through the use of behavioral strategies and self-management skills. Anthropometrical measurements and brief face-to-face counseling sessions were conducted by industrial health nurses at the beginning of the study and every 4 weeks throughout the intervention period. The average duration of the counseling sessions was between 20 and 30 minutes. Subjects were instructed to select special items from 20 suggestions for weight control (Table 3). To standardize the information given during the intervention, industrial health nurses were advised to use original educational tools. Between monthly counseling sessions, participants were supported by industrial nurses via a Web-based program (QUPiO, Heath Care Committee Ltd., Tokyo, Japan) that enabled remote but continuous intervention. The intervention tools and web-based programs both included figures and cartoons and offered practical information for weight and visceral fat reduction in a fun and enjoyable manner. 

### 3.4. Web-Based Weight-Loss Program Based on SCT

QUPiO was offered free of charge for use in the present study. It was designed based on Social Cognitive Theory (SCT), which focuses on the four following factors identified by Bandura as influencing self-efficacy: mastery experiences, social modelling, social persuasion, and both physical and emotional reactions. For example, a “mastery experience” within the web-based program consisted of participants setting their target weight and then selecting a special agenda of healthy activities that would enable them to achieve it. In a continuous process of self-regulation, participants were able to monitor their own physiological changes daily and record their achievements on a personal web page. To support establishment of “social modelling,” participants were given suggestions for 9 ideal lifestyle models, including “enjoying sports,” “wearing stylish clothes,” “going on a date with your spouse,” and others. Additionally, participants were able to obtain “social persuasion and emotional reactions” through participation in an online “chat room” with other participants.

### 3.5. Visceral Measurement and Awareness Program

Visceral fat area was estimated by bioelectrical impedance analysis (BIA) as reported previously [17]. Briefly, the voltage at the umbilicus position correlates significantly with visceral fat area and is negligibly influenced by subcutaneous fat; therefore, the area can be calculated based upon voltage. The correlation of BIA with the results from CT measurement was 0.88 [17].

After measurement of VFA, subjects received health counseling on the increased health risks associated with visceral fat obesity. By using 15 original educational leaflets, the subjects were guided to recognize subconscious issues that negatively influenced their dietary habits and physical activities. Another tool used was a booklet composed of 3 categories, success stories, and diet and exercise tips, each of which consisted of 101 agendas. This “101 × 3 booklet” was supplied to every subject in order to facilitate alternate selection of their “self-selected healthy activities” from 10 agendas on healthy eating habits and 10 agendas on physical activities (Table 3). Subjects were allowed to change these “self-selected healthy activities” if they found it difficult to incorporate them into their daily routine.

### 3.6. Statistical Analyses

Two analyses were performed on WC change and related outcome variables. The first was an intent-to-treat (ITT) analysis including all randomized participants, in which missing values were imputed by last-observation-carried-forward for participants who either did not satisfy inclusion criteria (BMI < 23) or deviated from the protocol. The second analysis was a completers-analysis limited to participants who completed the study.

Descriptive statistics were performed on the data collected in this study and continuous variables are presented as means ± standard deviation (SD). Categorical variables are presented as absolute numbers and percentages. Distributions were tested by Levene's test. Groups were compared by analysis of variance (ANOVA) or analysis of covariance (ANCOVA). Post hoc comparisons were conducted by either Tukey's HSD test or Dunnett's T3 test, depending on results from the distribution test (Levene's). Results are discussed based on a significance level of 5%  (*P* < 0.05). All analyses were performed using Statistical Package software (SPSS version 19 for Windows; SPSS, Chicago, IL, USA).

Statistical analyses were validated by a third party (Department of Medical Statistics, CIMIC Co., Ltd., Japan).

### 3.7. Assessment of Lifestyle Improvement

Achievement of readiness was assessed by a self-reported questionnaire at baseline and again at 12 weeks according to the following four stages reported in “Stages of Change Model” by Prochaska and DiClemente: (1) precontemplation, (2) contemplation, (3) preparation, and (4) action/maintenance. Achievement scores were calculated as follows: (1) count stages 1–3 as 0 and stage 4 as 1; (2) (sum of achievement scores, 12 weeks) − (sum of achievement scores, 0 week); (3) improvement scores were calculated by averaging the achievement scores in each group. 

## 4. Results

### 4.1. Baseline Characteristics

**Figure 1:**
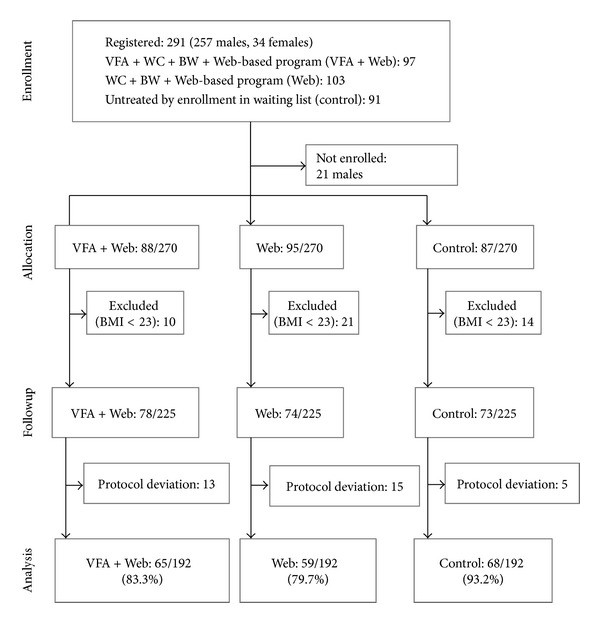
Schema of participants in this study. VFA: visceral fat accumulation, WC: waist circumference, BW: body weight, BMI: body mass index, and WEB: web-based program.

Of the 291 people assessed for eligibility in this study, 270 (236 males) were randomly allocated into the trial (Figure 1).  Table 1 shows the results of baseline measurements. Most of the participants were overweight (51, 21.6%) or obese (155, 65.7%).

### 4.2. Retention at 12 Weeks

Overall, the completion rate in this study was 85%. No significant difference in completion rate was observed among the control (68/73, 93.2%), Web only (59/74, 79.7%), and Web plus VFA (65/78, 83.3%) groups (Figure 1).

### 4.3. Changes in WC, Body Weight, and BMI

**Figure 2:**
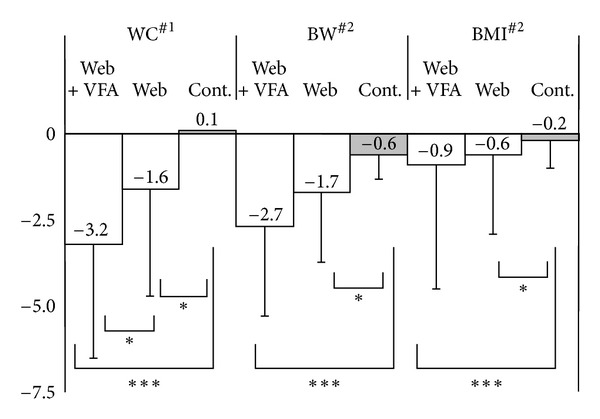
Significant decrease in waist circumference (WC, cm), body weight (BW, kg), and body mass index (BMI, kg/m^2^) in 192 completers. ^#^1: ANCOVA < 0.000; ^#^2: ANOVA < 0.000; *Tukey-HSD < 0.05; ***Tukey-HSD < 0.001.

Subjects in the Web and Web plus VFA groups showed significant reductions in WC and body weight, based on both BMI and kilograms lost (Table 2). WC decreased significantly more in the Web + VFA group than in the Web and control groups. A significant reduction in body weight was observed in the VFA + Web group compared to the control group. The decreases observed in both BW and BMI between groups were statistically significant according to analysis by ANOVA, while the decreases in WC were significant according to ANCOVA (Figure 2).

Of the 192 completers, the percentages of those who lost 5% or more of their initial body weight were 6%, 18%, and 30%, respectively, in the control, Web, and Web + VFA groups (ANOVA, *P* < 0.01). Post hoc comparisons showed that a significantly greater number of participants lost 5% of their initial body weight in the Web + VFA than in the control group (Tukey's HSD test, *P* < 0.01). The percentages of those who lost 5% or more of their initial WC were 13%, 12%, and 27%, in the control, WEB, and WEB + VFA group, respectively. 

### 4.4. Lifestyle Changes and Adverse Events

**Figure 3:**
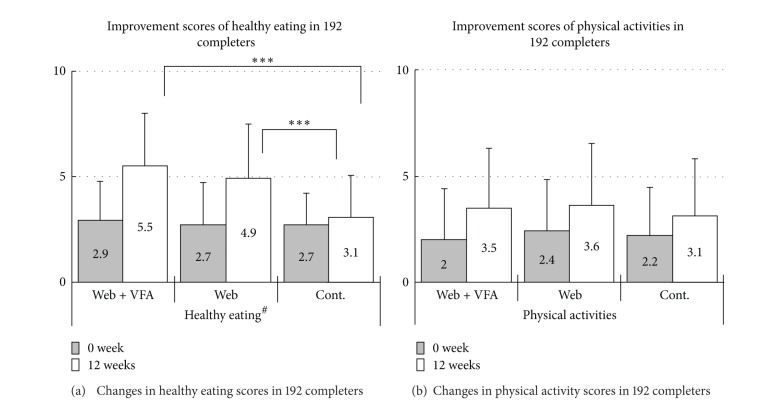
Achievement of readiness was assessed by a self-reported questionnaire at baseline and again at 12 weeks according to the following four stages reported in “Stages of Change Model” by Prochaska and DiClemente: (1) precontemplation, (2) contemplation, (3) preparation, and (4) action/maintenance. Achievement scores were calculated as follows: (1) count stages 1–3 as 0 and stage 4 as 1; (2) (sum of achievement scores, 12 weeks) − (sum of achievement scores, 0 week); (3) improvement scores were calculated by averaging the achievement scores in each group. ^#^ANOVA < 0.00; ***Tukey HSD < 0.00.

After 12 weeks, a statistically significant improvement in healthy diets in those randomly assigned to the Web only group compared to control (Figures 3(a) and Figure 3(b)) was seen. No adverse events were observed.

## 5. Discussion

### 5.1. VFA Program and Weight Loss

This study demonstrated that participating in either a Web-based weight-loss program alone or a Web program accompanied by information on visceral fat accumulation can lead to significant reductions in both WC and BW, variables that are of high clinical importance. These findings suggest that both programs lead to reductions in visceral fat, due to the fact that WC is a simple indicator of visceral fat accumulation. The weight loss in the Web and Web + VFA groups averaged about 2 to 3 kg (approximately 5% of initial body weight), and 15.9% of the randomized participants achieved weight loss of 5% or more. Few studies have examined the use of fully automated computer or internet programs for weight loss that do not involve human contact [18]. Supplementation of an internet-based weight-loss treatment with monthly in-person meetings did not result in greater weight loss over 12 months [19]. Micco et al. suggested that the dynamic, socially supportive, and interactive elements of websites may eliminate the need for further interpersonal behavioral counseling. Awareness and knowledge about weight status and management is also important [20]. The mass media campaign increased awareness of the link between obesity and cancer and the specific waist sizes indicative of risk and increased behavioral intentions with respect to weight and cancer. However, it did not have an effect on self-awareness of weight status, perceived personal risk of cancer, or weight loss behavior [21]. On the other hand, weight loss in the Web + VFA group averaged about 2.7 to 1.7 kg (approximately 3% of initial weight), and 15.9% of participants randomized into this group achieved weight loss of 5% or more. These findings suggest that a Web-based weight-loss program accompanied by a VFA program enhances weight loss through the promotion of healthy eating habits.

### 5.2. Limitations

There are a number of important potential limitations in the present study. First, the study population was predominantly male. This bias may limit the generalizability of our results. Second, since the duration of this study was short, we did not have data on weight maintenance and regain after the intervention. Third, we did not have laboratory measurements and therefore did not know the prevalence of metabolic syndrome before and after the intervention. Lastly, the small sample size limited the extent of additional analyses. Further research is needed to clarify these points.

## 6. Conclusion

A simple intervention that included measurement of VFA and brief counseling, in addition to a Web-based weight-loss program, promoted modest weight loss in workplace settings. Further examination that includes a longer study period is required to confirm these findings.

## Figures and Tables

**Table 1:** Baseline data of participants.

	WC (cm)	BW (kg)	BMI (kg/m^2^)
Web + VFA group (*n* = 88)	91.5 ± 8.6	75.3 ± 11.9	26.2 ± 3.2
Web group (*n* = 95)	89.4 ± 8.5	73.2 ± 12.0	25.6 ± 3.4
Control (*n* = 87)	88.1 ± 7.6	74.2 ± 9.8	25.4 ± 2.7

Total (*n* = 270)	89.7 ± 8.3	74.2 ± 11.3	25.7 ± 3.1

Web: Web-based program and VFA: visceral fat accumulation.

**Table 2:** Changes in waist circumference (WC, cm), body weight (BW, kg), and body mass index (BMI, kg/m^2^) according to study group.

Variables	Group	Completers (*n* = 192)		Intention to treat (*n* = 270)	
		Mean (SD)	Difference (CI versus control)	Mean (SD)	Difference (CI versus control)
WC (cm)	Web + VFA	−3.2 (3.3)	−3.3 (−4.5 to −2.1)	−2.7 (3.3)	−2.7 (−3.8 to −1.9)
	Web	−1.6 (2.6)	−1.6 (−2.8 to −0.5)	−1.3 (2.7)	−1.3 (−2.3 to −0.3)
	Control	0.1 (3.6)	—	0.0 (3.2)	—

BW (kg)	Web + VFA	−2.7 (3.1)	−2.0 (−3.0 to −1.1)	−2.2 (3.0)	−1.6 (−2.4 to −0.8)
	Web	−1.7 (2.0)	−1.0 (−1.8 to −0.3)	−1.3 (0.6)	−0.5 (−1.5 to +0.5)
	Control	−0.6 (2.3)	—	−0.6 (2.2)	—

BMI (kg/m^2^)	Web + VFA	−0.9 (1.1)	−0.8 (−1.1 to −0.4)	−0.8 (1.0)	−0.6 (−0.9 to −0.3)
	Web	−0.6 (0.7)	−0.4 (−0.6 to −0.1)	−0.5 (0.7)	−0.3 (−0.5 to +0.0)
	Control	−0.2 (0.8)	—	−0.2 (0.7)	—

Web: web-based program; VFA: visceral fat accumulation.

SD: standard deviation; CI: confidence interval.

**Table 3:** Agendas for lifestyle improvement.

	Dietary habits	Physical activities
1	Do not overeat (eat too much)	Being physically active on holidays
2	Chew well and eat slowly	Increase daily walking (shopping and commuting)
3	Avoid sweet beverages and canned coffee that contains sugar	Use stairs instead of escalator or elevator
4	Cut down on between-meal snacks	Increase opportunities for exercise and stretching
5	Reduce consumption of sweet buns, pastries, and rolls	Always place walking shoes at the entrance of my house
6	Consume many vegetables (3 times or 5 servings per day)	Wear the pedometer everyday
7	Avoid noodles and rice in the same meal	Walk 30 minutes, twice per week
8	Restrain from eating deep fried dishes more than 3 times per week	Walk more than 10,000 steps (count by pedometer)
9	Drink moderately (<20 g of alcohol per day)	Do resistance training 3 times per week
10	Avoid eating at night	Do some “sports” once a week

Assessment of lifestyle improvement.

Achievement of readiness was assessed by a self-reported questionnaire at baseline and again at 12 weeks, according to the following four stages: (1) precontemplation, (2) contemplation, (3) preparation, and (4) action/maintenance.
